# Compensative Resistance to Erastin-Induced Ferroptosis in GPX4 Knock-Out Mutants in HCT116 Cell Lines

**DOI:** 10.3390/ph16121710

**Published:** 2023-12-10

**Authors:** Malgorzata Adamiec-Organisciok, Magdalena Wegrzyn, Lukasz Cienciala, Damian Sojka, Joanna Nackiewicz, Magdalena Skonieczna

**Affiliations:** 1 Department of Systems Engineering and Biology, Faculty of Automatic Control, Electronics and Computer Science, Silesian University of Technology, Akademicka 16, 44-100 Gliwice, Poland; 2 Biotechnology Centre, Silesian University of Technology, Krzywoustego 8, 44-100 Gliwice, Poland; 3 Student Science Club of Engineering and Systems Biology, Biotechnology Centre, Silesian University of Technology, Krzywoustego 8, 44-100 Gliwice, Poland; 4 Maria Skłodowska-Curie National Research Centre and Institute of Oncology, Gliwice Branch, Wybrzeze Armii Krajowej 15, 44-102 Gliwice, Poland; 5 Faculty of Chemistry, University of Opole, Oleska 48, 45-052 Opole, Poland

**Keywords:** glutathione peroxidase GPX4, ferroptosis cell death, ferroptosis-resistance, ferroptosis-sensitiveness, CRISPR/Cas-9 technique

## Abstract

Ferroptosis results from the accumulation of oxidized and damaged lipids which then leads to programmed cell death. This programmed process is iron-dependent, and as a fundamental biological process, plays a crucial role in tissue homeostasis. The ferroptosis molecular pathway depends on self-regulatory genes: GPX4; TFRC; ACSL4; FSP1; SLC7A11, and PROM2. Some of them were considered here as ferro-sensitive or ferro-resistance markers. We examined the impact of GPX4 gene knock-out, using the CRISPR/Cas-9 technique, on ferroptosis induction in the HCT116 colorectal cancer cell line. The results confirmed that cells lacking the GPX4 gene (GPX4 KO) should be more susceptible to ferroptosis after erastin treatment. However, the decrease in cell viability was not as significant as we initially assumed. Based on the lipid peroxidation markers profile and RT-qPCR gene expression analysis, we revealed the activation of an alternative antioxidant system supporting GPX4 KO cells, mostly for cellular ferroptotic death avoidance. Increased expression of FSP1 and PRDX1 genes in knock-out mutants was associated with their function—recognized here as ferroptosis suppressors. For such reasons, studies on the role of GPX4 and other crucial genes from the ferroptotic pathway should be explored. Despite promising prospects, the utilization of ferroptosis mechanisms in cancer therapy remains at the stage of experimental and in vitro preclinical studies.

## 1. Introduction

Glutathione peroxidase 4 (GPX4) is an antioxidant enzyme belonging to the family of glutathione peroxidases. GPX4 catalyzes the reduction of hydrogen peroxide, organic hydroperoxides, and lipid hydroperoxides at the expense of reduced glutathione and protect cells against oxidative stress. GPX4 differs from other GPX family members with its monomeric structure, less dependence on glutathione as a reducing substrate, and its ability to reduce lipid hydroperoxides within biological membranes [1]. Glutathione (GSH) biosynthesis and proper GPX4 function are crucial for ferroptosis control, and GPX4 inhibition can increase cell sensitivity to ferroptosis [2]. After cystine is absorbed by the X_c_^−^ system, it is reduced to cysteine for GSH synthesis, which sustains GPX4 activity [3].

**Figure 1:**
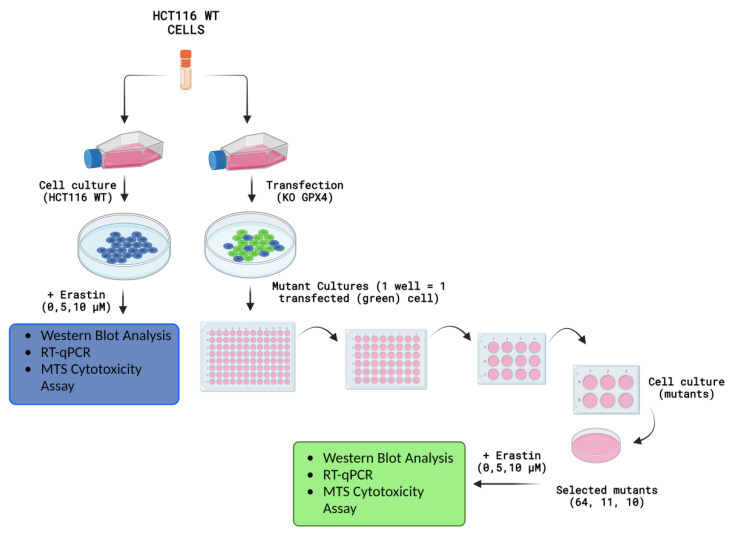
Experiments and measurements conducted on HCT116 WT, GPX4 KO (clone No’s. 10 and 11), and positive control (clone No. 64) cell lines after erastin exposure. Created with Biorender.com.

Despite breakthroughs in the field of anticancer therapy, cancer remains the second leading cause of death worldwide [4,5]. Currently, the primary therapeutic approach for chemotherapy involves the use of anticancer drugs to induce apoptotic cell death in cancer cells. However, due to the inherent and acquired resistance of cancer cells to apoptosis, their therapeutic effect is limited. Drug resistance remains a major limiting factor for the successful treatment of cancer patients [6]. The induction of ferroptosis in cancer cells is one of the most effective ways to overcome drug resistance. There are several ways to achieve this using exogenous molecules or drugs or by regulating physiological conditions in the external environment (e.g., a high extracellular glutamate concentration), thereby blocking the X_c_^−^ system [7,8,9]. Another form of anticancer therapy is radiotherapy, which utilizes ionizing radiation (IR) from a radioactive source to induce DNA damage and apoptosis in cells [10]. Studies have reported that ferroptosis inducers (such as RSL3, erastin, sorafenib, and sulfasalazine) can enhance the effectiveness of radiotherapy by inhibiting SLC7A11 or inactivating GPX4 in tumor models such as glioma, lung cancer, fibrosarcoma, melanoma, breast cancer, and cervical cancer [9,11,12,13,14]. In addition to reducing SLC7A11 levels, radiotherapy also increases ACSL4 levels, thus enhancing lipid synthesis and peroxidation ultimately resulting in the induction of ferroptosis [11]. Anticancer therapies, based on the induction of oxidative stress, ionizing radiation or photo-sensitizer addition, also result in cellular macromolecule (e.g., DNA, lipids) oxidation [15]. Ferroptosis is regulated by many genes, including p53, GPX4, TFRC (transferrin receptor), SLC7A11 (solute carrier family 7 Member 11), and ACSL4 (acyl-CoA synthetase long-chain family member 4). This process mainly involves genetic alterations in iron homeostasis and lipid peroxidation metabolism [16,17]. Cancer cells’ resistance to ferroptosis induction, together with apoptosis avoidance and oxidative stress reduction, makes them unpredictable to chemo- or radiotherapy protocols. The phenomenon of chemoresistance to erastin-induced ferroptosis remains undiscovered, even after genome editing manipulation with protective gene knock-out. For such purposes, the aim of this study was to use the HCT116 WT colon cancer cell line for genome editing using the CRISPR/Cas-9 technique in order to knock-out the glutathione peroxidase 4 (GPX4) gene, and to investigate how this process would affect the induction of regulated cell death (ferroptosis) and other cellular processes (Figure 1).

## 2. Results

### 2.1. Evaluation of CRISPR/Cas-9 Knock-Out for GPX4 by Western Blot

**Figure 2:**
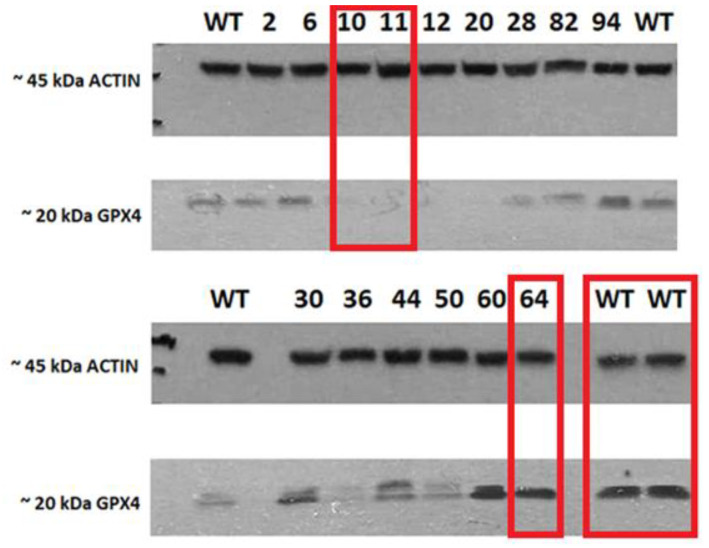
Expression of GPX4 and actin in cell lysates. The red rectangle highlights the sample (clones with mutations (No. 10 and 11), positive (64), and wild-type (WT) variants of the HCT116 cell line that were selected for further analysis.

The characterization of HCT116 cells by the Western blot method revealed the full expression of the GPX4 protein in mutant No. 64 (GPX4 positive control) and the untreated control HCT116 (WT). No noticeable GPX4 protein expression was observed in mutants’ No’s. 10 and 11 (GPX4 KO). Actin was used as a standard for protein expression and for ensuring the equal loading of proteins onto the gel (Figure 2).

The PCR amplicons with the shortened sequence of GPX4 are presented in the Supplementary File Figure S1.

Following the knock-out control of the GPX4 gene by Western blot, the following mutants were chosen for further analysis in the study: No. 64 (as a positive control for the positive GPX4 variant), No’s. 10 and 11 (GPX4 KO cell lines, confirmed by Western blot). In addition to the mentioned mutants, an untreated control was also used; the wild-type cell line (HCT116 WT), which was not subjected to genome modifications (Figure 2). PCR amplicons with a shortened sequence of GPX4 are presented in the Supplementary File Figure S1 and the original Western blot visualized in Figure S6.

### 2.2. Cell Viability after Ferroptosis Induction

For the three selected mutants, No’s. 10, 11 and 64, and the HCT116 WT control line, an MTS assay was performed to determine mitochondrial activity and cell viability after pre-treatment with erastin. The first MTS test was conducted to determine the IC_50_ values, allowing for the selection of appropriate doses (5 and 10 µM) of erastin for further analysis. HCT116 WT and GPX4 KO cells responded to the dose of erastin at 10 µM, within the determined IC_50_ values (Table 1).

### 2.3. Changes in the Expression Levels of Ferroptotic Pathway Marker Genes

#### 2.3.1. Changes in the Expression Levels of the Inductors and Protectors against Ferroptosis, ACSL4 and GPX4 Genes

**Figure 3:**
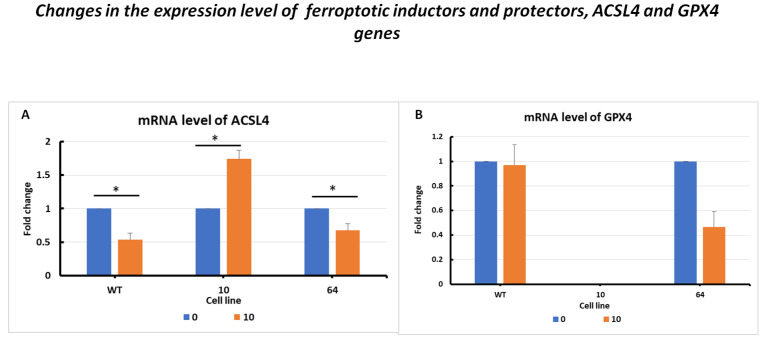
mRNA levels of ACSL4 (**A**) and GPX4 (**B**) in HCT116 WT cells and mutants No. 64 (GPX4 positive control) and 10 (GPX4 KO) 24 h after erastin addition. Blue bars—untreated control; orange bars—10 µM erastin. Values are presented as the mean ± standard deviation. Statistical significance is indicated by “*” for values where *p* < 0.05.

The changes in the expression level of the ACSL4 gene showed overstimulation after erastin exposure at a higher dose of 10 µM in GPX KO mutant No. 10, which is a typical response to positive stimulation (Figure 3A). CRISPR/Cas-9 manipulation decreased the expression of observed GPX4 KO cells—the amplicons for the GPX4 gene were not detected in clone No. 10 (Figure 3B). Thus, the downregulation of GPX4 genes in sensitivity to ferroptosis cell lines resulted in the overexpression of death pathway executors, with the ACSL4 pro-oxidative enzyme.

The results showed changes in the GPX4 gene expression levels in the selected HCT116 cell mutants. In the case of HCT116 WT cells, there was a slight difference between the erastin-treated and untreated controls. After the erastin treatment of mutant No. 64 cells, a decrease in GPX4 gene expression was observed compared to that in the control. However, for mutant No. 10, no GPX4 expression product was observed after erastin treatment.

#### 2.3.2. Changes in the Expression Levels of Intra- and Extracellular Iron Transporters, TFRC and FSP1 Genes

**Figure 4:**
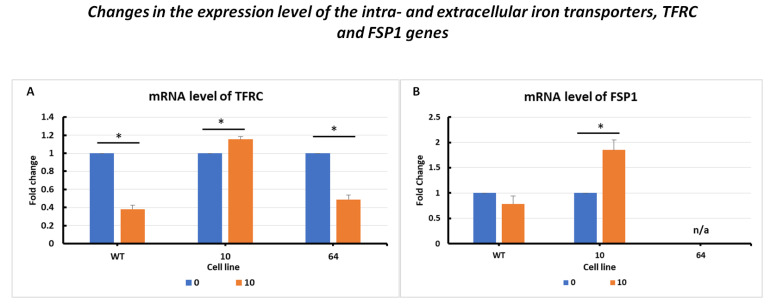
mRNA level of TFRC (**A**) and FSP1 (**B**) genes in HCT116 WT cells and mutants No. 64 (GPX4 positive control) and 10 (GPX4 KO) 24 h after erastin addition. Blue bars—untreated control; orange bars—10 µM erastin. Values are presented as the mean ± standard deviation. Statistical significance is indicated by “*” for values where *p* < 0.05.

The changes in TFRC mRNA expression levels (Figure 4A) are presented for the three selected cell lines: HCT116 WT, mutant No. 64 (GPX4 positive control), and mutant No. 10 (GPX4 KO). Differences in TFRC expression were observed, with a correlation with GPX4 gene status. In the case of HCT116 mutant No. 10 (GPX4 KO) cells, overexpression was noticeable when erastin was applied (at a dose of 10 µM) compared to that in the untreated control. For HCT116 WT and mutant No. 64 cells, a decrease in expression was observed after the addition of erastin.

Analysis of changes in FSP1 gene expression levels (Figure 4B) showed a decrease in expression in the case of HCT 116 WT cells subjected to erastin compared to the untreated control. In cells in which the GPX4 gene was deleted (HCT116 mutant No. 10), a significant increase in FSP1 expression was observed after erastin treatment.

#### 2.3.3. Changes in the Expression Level of the Antioxidant System, PRDX1 and TRX Genes

**Figure 5:**
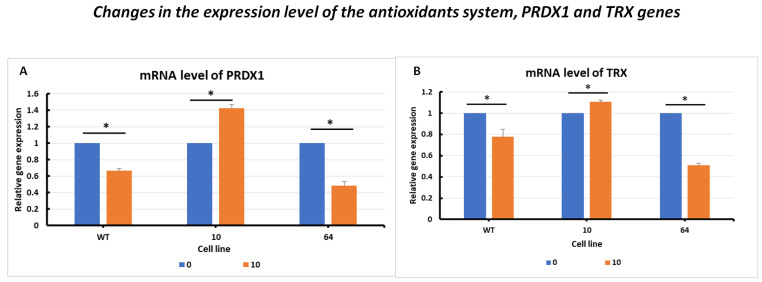
mRNA levels of PRDX1 (**A**) and TRX (**B**) genes in HCT116 WT cells and mutants No. 64 (GPX4 positive control) and 10 (GPX4 KO) 24 h after erastin addition. Blue bars—untreated control; orange bars—10 µM erastin. Values are presented as the mean ± standard deviation. Statistical significance is indicated by “*” for values where *p* < 0.05.

The changes in the expression level of the peroxiredoxin PRDX1 gene (Figure 5A) showed activation after erastin addition. Significantly elevated PRDX1 expression levels were observed in GPX4 KO mutant No. 10 cells treated with erastin. For the other cell lines, WT and mutant No. 64 (GPX4 positive control), a decrease in PRDX1 levels was observed in samples treated with erastin compared to the control samples, indicating a negative correlation between GPX4 gene status and PRDX1 expression.

In the analysis of changes in the expression level of the thioredoxin TRX gene (Figure 5B), a similar expression profile to that of PRDX1 was observed in WT and GPX4 KO cells. A negative correlation between the GPX4 gene status and TRX expression was evident.

#### 2.3.4. Changes in the Expression Level of Extracellular Small Vehicle Production, PROM1 and PROM2 Genes

**Figure 6:**
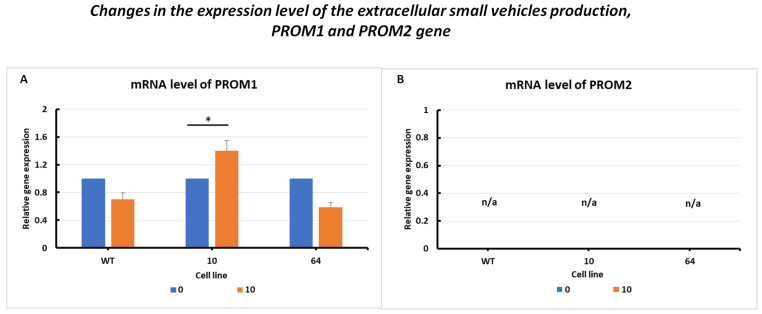
mRNA levels of PROM1 (**A**) and PROM2 (**B**) genes in HCT116 WT cells and mutants No. 64 (GPX4 positive control) and 10 (GPX4 KO) 24 h after erastin addition. Blue bars—untreated control; orange bars—10 µM erastin. Values are presented as the mean ± standard deviation. Statistical significance is indicated by “*” for values where *p* < 0.05.

The results of changes in PROM1 gene expression levels (Figure 6A) are presented with similar overexpression in GPX KO cells. In the HCT116 WT and mutant No. 64 (GPX4 positive control) cell lines, it was observed that PROM1 gene expression decreased upon erastin treatment compared to the untreated control. In mutant No. 10 cells, a significant increase in PROM1 expression was observed following the addition of erastin.

PROM2 gene expression was observed in all the examined cell lines (Figure 6B). This result is consistent with the findings in the literature, where PROM2 is considered a marker of resistance to ferroptosis. An increase in the expression of this gene was observed in resistant cell lines, whereas a decrease or absence of expression was observed in sensitive cell lines. The colorectal cancer HCT116 cell line was found to be sensitive to ferroptosis cell death, with a silenced PROM2 gene (Figure 6B). 

The cells resistant to ferroptosis, so called positive control, with high expression of PROM2 gene is presented as an example in Supplementary File, Table S2. 

### 2.4. Correlations between Genes

**Figure 7:**
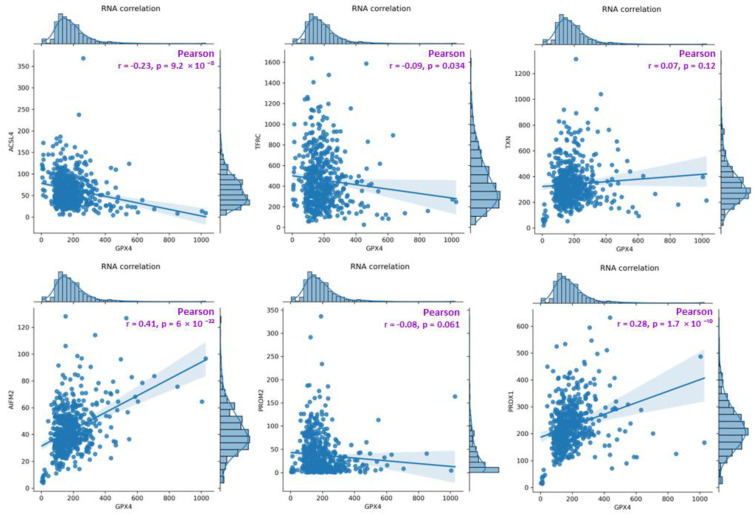
A diagram presenting a summary of correlations between GPX4 RNA levels and the RNA levels of the analyzed genes (**top**: ACSL4, TFRC, TXN(TRX); **bottom**: FSP1(AIFM2), PROM2, PRDX1). The analysis was performed on data obtained from the FerrDB database, TCGA-COAD (The Cancer Genome Atlas—Colon adenocarcinoma, FerrDb V2; [18]).

Based on the FerrDb V2 [18] online database, a general summary of the correlations (Figure 7) between GPX4 RNA levels and the RNA levels of the analyzed genes was generated (top: ACSL4, TFRC and TXN (TRX); bottom: FSP1 (AIFM2), PROM2 and PRDX1). For TFRC, ACSL4, and PROM2, a weak negative correlation is observed (the coefficient “r” falls within the range from −1 to −0.5) with respect to GPX4, and the results for TFRC and ACSL4 are statistically significant (*p*-value < 0.05). For the other genes (TXN (TRX), FSP1 (AIFM2) and PRDX1), a positive correlation with GPX4 was observed. For TXN, the correlation was very weak and not statistically significant (*p*-value > 0.05). The strongest correlation among all the genes was observed for FSP1 (AIFM2). RNA level data were acquired and visualized within the FerrDb V2 database (additional visualization for correlation analysis is presented in the Supplementary File Figures S3–S5). 

## 3. Discussion

### 3.1. Sensitiveness to Ferroptosis Induction upon GPX4 Status in HCT116 Cell Lines

The presented work, focused on the cellular death pathway of ferroptosis, was based on CRISPR/Cas-9 cellular model creation for mutant cell lines with GPX4 gene knock-out. In the validation process of mutants with a silenced GPX4 gene and lacking in functional protein production, by Western blot (Figure 2), two clone cell lines were selected (mutants No. 10 and 11). Mutant No. 64 was used as a positive control, which, despite the same procedure, still contained the specified gene and protein production ability. The selected clones were stimulated with erastin to induce ferroptotic death (Figure 1).

Ferroptosis was induced by erastin at doses determined based on the calculated IC_50_ values (Table 1) and the cell lines were characterized in terms of ferroptotic cell death. Analysis of cell viability using the MTS assay (Table 1) showed that mutant cell line No. 10 (GPX4 KO) was the most susceptible to erastin (at a dose of 10 µM), whereas cell lines with a positive GPX4 gene status (the WT line and mutant No. 64, the GPX4 positive control) were the least susceptible, which is consistent with the hypothesis proposed in the study and the scientific literature [11,12,13,19]. Additional visualization of lipid peroxidation as a sign of ferroptosis is presented in the Supplementary File Figure S2**.**

Mutant No. 10 GPX4 KO treated with erastin at a dose of 10 µM showed the overexpression of all analyzed marker genes (TFRC, ACSL4, FSP1, TRX, PRDX1, and PROM2), except for PROM2 and GPX4, for which no primer amplification was obtained (Figure 3, Figure 4, Figure 5 and Figure 6). PROM2 is considered to be a ferroptosis suppressor gene due to its ability to induce resistance against this type of cell death. Previous studies have shown that PROM2 affects the stimulation of exosomal iron export and the formation of multivesicular bodies containing ferritin, which in turn leads to reduced lipid peroxidation and, consequently, resistance to ferroptosis [19]. In our sensitive HCT116 WT and GPX4 KO mutated cell lines, the lack of PROM2 expression at the mRNA level (Figure 6B) may result from the sensitivity of colorectal cancer cell lines to ferroptotic cell death, similar to previously reported findings. Literature reports and our results suggest that PROM2 expression may be a good indicator of cellular sensitivity to ferroptosis. Reduced or absent expression of this gene is observed in sensitive lines, whereas its overexpression is observed in resistant lines [20,21].

Conversely, the reduced expression of GPX4 may confirm successful excision of the gene fragment during genome editing using CRISPR/Cas-9 (Figure 3B). The expression of ferroptotic marker genes resulted in opposite expression profiles. Differences were observed between the wild-type (WT) and No. 64 (GPX4 positive control) lines with a decrease or lack of expression observed for all of the specified genes in GPX4 KO mutants (Figure 3, Figure 4, Figure 5 and Figure 6). The expression level of PROM1 (Figure 6A), which is proposed as a biomarker for extracellular vesicles in colorectal cancer production, was also examined and showed the stimulation of the iron export system.

### 3.2. Alternative Antioxidant System upon Erastin Regulation

Peroxiredoxin 1 (PRDX1) is a member of the widely occurring thioredoxin peroxidase family, which catalyzes the reduction of peroxides, including hydrogen peroxide. It functions as an antioxidant enzyme similar to glutathione peroxidase (GPX). Scientific literature reports that PRDX1 is essential in cells and plays an important role in maintaining the homeostasis of reactive oxygen species within cells (or in the precise regulation of cellular ROS levels) [22]. Besides GSH, thioredoxin (TXN) is the second most significant antioxidant. In some malignant tumors, the TXN-dependent system is often activated, and the simultaneous inhibition of both GSH and TXN pathways is an effective method for inducing cell death [23,24]. In the tested cell lines, WT and GPX4 KO, the antioxidant alternative system was activated after erastin addition (Figure 5).

### 3.3. Iron Homeostasis for Intracellular Ferroptosis Execution

The results obtained from the FerrDB database [18] are presented in the form of correlation charts between the GPX4 RNA concentration and the RNA concentration of the analyzed gene (Figure 7). The weakest negative correlations were observed for TFRC, ACSL4, and PROM2. GPX4 is a protein that protects cells from ferroptotic cell death, while TFRC and ACSL4 drive ferroptosis by transferring iron from outside the cell to its interior and activating polyunsaturated fatty acids (PUFA), respectively [9,13]. Weak negative correlations for PROM2 in tissue data for colorectal cancer confirmed the hypothesis of the sensitivity of colorectal cancer cell lines. The strongest positive correlations among all the genes analyzed were found for PRDX1 and FSP1 (AIFM2), whereas the weakest was found for TXN (Figure 5). FSP1 acts in parallel with GPX4 as an inhibitor of phospholipid peroxidation, thereby inhibiting peroxidation and preventing ferroptosis [25]. The scientific literature reports that in the absence of GPX4, FSP1 can fully counteract lipid peroxidation and, consequently, ferroptosis [25,26].

Iron is an essential element in the fluidics of the body and is crucial for extra- and intracellular processes. Its improper distribution and concentration can affect and disturb physiological processes [27]. Iron has been known to play an important role in ferroptosis for several years:

- During ferroptosis induction, an increase in cellular labile iron is typically observed [28];
- The generation of reactive oxygen species (ROS) via iron in the Fenton reaction promotes lipid peroxidation in ferroptosis [27,29];
- Excess heme and non-heme iron can directly induce ferroptosis [30];
- Iron increases cell sensitivity to ferroptosis inducers (e.g., erastin) [29];
- Iron chelators block ferroptotic cell death in vitro and in vivo [29];
- Several heme and non-heme iron-containing enzymes, such as ALOX, NOX, and CYP, are responsible for lipid peroxidation [31].

## 4. Materials and Methods

### 4.1. Cell Culture

The HCT116 wild-type (WT) colorectal cancer cell line, obtained from the American Type Culture Collection (ATCC), and modified by CRISPR/Cas-9 genome editing, was used in this study. The GPX4 gene was deleted in cells referred to as “GPX4 KO” or “mutants”. An additional procedure, subjected to the CRISPR/Cas-9 without genome modification, was performed for the “GPX4 positive control” cell line. Cells were cultured in DMEM/F12 medium (Pan-Biotech, Aidenbach, Germany) supplemented with 10% fetal bovine serum (FBS; EURx, Gdansk, Poland) and penicillin–streptavidin solution (100×; Merck, Darmstadt, Germany). The cell culture was performed under standard conditions. The cells and HCT116 mutants were evaluated using protocols and experimental in vitro procedures after 24 h of erastin exposure (Figure 1).

### 4.2. MTS Cytotoxicity Assay

Cells were seeded into a 96-well plate at a density of 8000 cells per well. After 24 h, the medium was removed from the wells, and various doses of erastin (0, 5, 10, 25, 50, and 75 μM) were added. The plate was incubated for the next 24 h. After this time, the cell culture medium was removed from the wells, and 7 μL of a solution of yellow 3-(4,5-dimethylthiazol-2-yl)-5-(3-carboxymethoxyphenyl)-2-(4-sulfophenyl)-2H-tetrazolium, also known as MTS salt (reagent MTS, #G109A, Promega, Madison, USA), and 43 μL of colorless DMEM/F12 cell culture medium was added. After 20 min, the absorbance was measured at a wavelength of 490 nm using a microplate spectrophotometer (BioTek Epoch 2; Winooski, VT, USA).

#### 4.2.1. IC_50_ Determination

Cells were treated with various doses of erastin to determine the half-maximal inhibitory concentration (IC_50_) of erastin in each of the tested cell lines. IC_50_ is the concentration of a compound that reduces cell survival by 50% compared that of the untreated control. The summary table of calculated IC_50_ values for the HCT116 cell lines treated with erastin was supplemented with cell survival graphs, created using Microsoft Excel 2010.

After analyzing the IC_50_ values, two doses of erastin were selected for further experiments: one at a dose close to the IC_50_ value for most tested cell lines (10 µM erastin) and the other at half of that dose (5 µM erastin). Subsequently, cell viability was assessed again using the MTS assay. A 96-well plate was seeded, as previously described. After 24 h, the erastin solution was added to the cells in two doses: 5 µM and 10 µM. Cell viability was evaluated after 24 h by adding the MTS reagent (yellow tetrazolium). The results were presented as the survival fraction, SF [%] from the untreated controls.

#### 4.2.2. Modification of HCT116 Cell Lines

To create the CRISPR/Cas9 modified HCT116 cell line, the following procedure was employed: initially, cells were seeded in 12-well plates at a density of 80 × 10^4^ cells per well. The following day, transfection was performed using 1 μg of the glutathione peroxidase 4/GPX4 Double Nickase Plasmid (sc-401558-NIC, Santa Cruz Biotechnology, Inc., Dallas, TX, USA) with Lipofectamine 3000 (Life Technologies, Carlsbad, CA, USA). Transfected cells were then cultured in selective DMEM/F12 medium supplemented with puromycin (1 µg/mL; Merck KGaA, Darmstadt, Germany). Single isogenic clones were created by limiting dilution in 96-well plates. Alterations in the GPX4 gene within these clones were assessed via Western blot (WB) and RT-qPCR. Ultimately, two knock-out cell lines, HCT116_KO_10 (clone No. 10) and HCT116_KO_11 (clone No. 11), with complete GPX4 gene excision, were obtained. Additionally, HCT116_ctr_64 (clone No. 64) served as the positive control, having undergone the transfection process, but retaining the intact gene.

#### 4.2.3. Western Blot CRISPR/Cas-9 Mutant’s Protein Detection

Western blot analysis is a commonly used technique to quantify specific proteins. To prepare total cellular protein extracts, cells were seeded in 6 cm dishes at a maximum confluence of 70%. After 24 h, the total protein extracts were collected by scraping the cells in RIPA buffer (Eurx, Poland, and 1 mM PMSF) supplemented with a mixture of 1x protease inhibitors (Roche Molecular Systems, Inc; Rotkreuz, Switzerland). The samples were incubated on ice for 15 min, and the lysates were centrifuged (4 °C for 20 min at 22,000× g). Protein concentration was determined using a Protein Assay Kit (Bio-Rad; Hercules, CA, USA) according to the manufacturer’s instructions. Equal amounts of protein, for each sample were separated by SDS-PAGE on 12% polyacrylamide separating gels and transferred to a nitrocellulose membrane using a Trans Blot Turbo system (Bio-Rad; Hercules, CA, USA) for 10 min. Membranes were blocked in 5% skim milk/TTBS (0.25 M Tris–HCl (pH 7.5), 0.15 M NaCl, and 0.1% Tween-20) for 1 h. Antibody–antigen interactions were detected using secondary antibodies (Table 2) and visualized using WesternBright Quantum HRP substrate (Advansta; San Jose, CA, USA). X-ray films (Carestream Health, Inc, Rochester, NY, USA) were used to detect chemiluminescent signals. β-Actin was used as a protein control.

#### 4.2.4. Real-Time qPCR for Marker Genes Evaluation

The day after sowing, the cells were exposed to medium containing the ferroptosis inducer—erastin (Sigma) for 24 h. Cells were harvested, and total RNA was isolated using a total RNA isolation kit (A&A Biotechnology, Gdansk, Poland) according to the manufacturer’s protocol. cDNA synthesis was performed using the NG-dART kit (EURx, Poland) in accordance with the protocol, and RT-qPCR reactions were performed using the RT PCR Mix SYBR^®^ kit (A&A Biotechnology) in accordance with the manufacturer’s protocol. Death marker genes were selected, primers were designed and the sequences, along with the annealing temperature, are shown in Table 3. The thermocycler was operated and the data analysis was performed using Bio-Rad CFX Maestro 1.1 software (Bio-Rad). RPL41 served as a reference gene, indicating that it acted as an expression standard for the studied genes with constant expression. The reaction was conducted according to the following protocol: initiation 2 min 50 °C; matrix denaturation and hot starting of polymerase 4 min 95 °C; PCR cycles No. 45 of: 45 s 95 °C, 30 s (annealing temperature according to Table 3), 30 s 72 °C; amplicons synthesis was finished with 5 min at 72 °C; melting curve assessed by temperature increase from 57 °C to 92 °C (every 0.5 °C).

#### 4.2.5. RT-qPCR and Data Statistical Analysis

The recorded data, the values of the cycle threshold Cq (Ct), were used to calculate the relative expression levels of the tested genes in the treated (erastin-exposed) cells compared to the untreated (control) cells. To calculate the relative increase in expression, Livak’s method, R = 2^−ΔΔCt^, was employed [32]. The measured values included both the reference gene (RPL41) and genes relevant to the ferroptotic cell death pathway (Table 3). The PCR reaction used for GOX4 KO validation is described in the Supplementary File Figure S1.

All results were presented as the means of at least three independent biological replicates. The significance of the level of changes in the tested samples, compared to the control sample (not exposed to erastin), was assessed using Student’s *t*-test. Statistical significance was denoted as “*” at *p* < 0.05 using Microsoft Excel 2010. Calculations are added in Table S1 (see Supplementary File).

## 5. Conclusions

In summary, the conducted study confirmed the main hypothesis of a decrease in the viability of GPX4 KO cells after treatment with an erastin dose close to the determined IC_50_ value. However, this decrease was not as significant as initially assumed. Lipid peroxidation analysis did not reveal peroxidation in any of the analyzed lines). Microscopic observations revealed a significant increase in reduced lipids that was positively correlated with the erastin dose, indicating the activation of an antioxidant system other than the glutathione shield, which is a feature of resistance to ferroptosis in cell lines [33]. Among the genes characterized by high expression in the GPX4 KO line, FSP1 and PRDX1 were notable for their known ferroptosis-suppressive properties. By analyzing the obtained data and scientific literature, it can be inferred that GPX4 KO cells avoided lipid peroxidation owing to the antioxidative action of FSP1.

These results emphasize the need for further analysis of the GPX4 gene’s function and other expressed genes in the ferroptosis process to better understand the biology of the tumor microenvironment. Despite promising prospects, the use of the ferroptosis mechanisms described in this study for cancer therapy remains at the stage of experimental preclinical research. Further research is necessary to better understand these mechanisms and develop effective and safe therapies based on modifications of the ferroptosis pathway. The expression profile of ferroptotic pathway genes showed that resistance to erastin exposure is independent of GPX4 knock-out in HCT116 colorectal cell lines.

## Figures and Tables

**Table 1:** IC_50_ values calculated from MTS assay and cell survival graphs. Data are presented as the mean ± standard deviation from 3 experiments. Graphs made using Microsoft Excel 2010.

Cell Line	IC_50_ [µM]	Cell Survival Graphs
HCT116 WT	10.46 ± 0.32	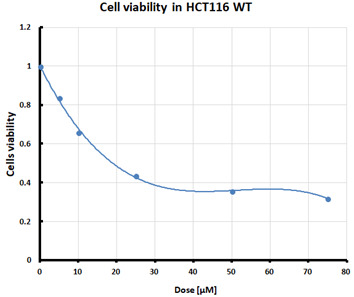
HCT116 positive control (mutant No. 64)	18.48 ± 4.36	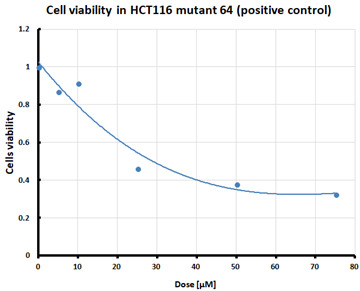
HCT116 GPX4 KO (mutant No. 10)	10.85 ± 2.58	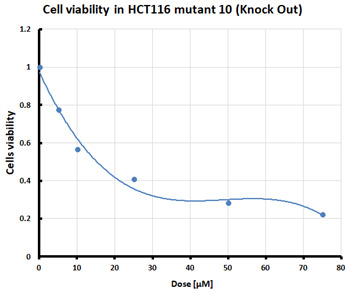
HCT116 GPX4 KO (mutant No. 11)	11.12 ±1.05	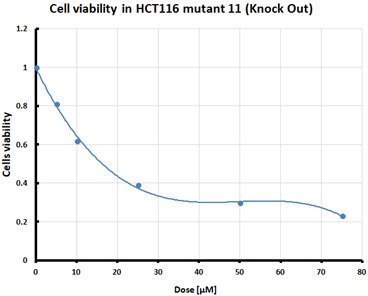

**Table 2:** List of Western blot reagents and primary and secondary antibodies.

Primary Antibodies					
Lp.	Antibody	Organism	Manufacturer	Used Dilution	Antibody Dilution Solution
1	anti-GPX4	rabbit	Proteintech	1:500	5% milk solution
2	anti-β-actin	rabbit	Sigma-Aldrich	1:5000	5% milk solution
**Secondary Antibodies**					
1	anti-rabbit	mouse	Santa Cruz Biotechnology	1:20,000 for GPX4 detection, 1:10,000 for β-actin detection	5% milk solution

**Table 3:** Temperatures used in the qPCR reaction and primer sequences.

Gene ID	Temperature [°C]	Forward Primer Sequence	Reverse Primer Sequence
TFRC	57	5′ GGAGACTGTCCCTCTGACTGG 3′	5′ GCTTCACATTCTTGCTTTCTGAG 3′
ACSL4	57	5′ GCTATCTCCTCAGACACACCGA 3′	5′ AGGTGCTCCAACTCTGCCAGTA 3′
GPX4	63	5′ AGTGAGGCAAGACCGAAGTAA 3′	5′ CTTCCCGAACTGGTTACACG 3′
PROM1	60	5′ TCCACAGATGCTCCTAAGGC 3′	5′ GCGGCTGTACCACATAGAGA 3′
PROM2	60.2	5′ AGAGCACCTGACATTCACCC 3′	5′ CTCGTACCGCACCACCTCAT 3′
FSP1	62	5′ CTGCCCTTCTCTCATCTTATCCT 3′	5′ CTGCCTCACCATGTCCTCATAG 3′
TRX	58	5′ TGAAGCAGATCGAGAGCAAGAC 3′	5′ TTCATTAATGGTGGCTTCAAGC 3′
PRDX1	54.4	5′ TCCTTTGGTATCAGACCCGA 3′	5′ GAGATGCCTTCATCAGCCTTT 3′
RPL41	57–62	5′ TCCTGCGTTGGGATTCCGTG 3′	5′ ACGGTGCAACAAGCTAGCGG 3′

## Data Availability

Data are contained within the article and supplementary material.

## Supplementary Materials

The following supporting information can be downloaded at: https://www.mdpi.com/article/10.3390/ph16121710/s1, Figure S1. Procedure of GPX4 KO validation in HCT116 WT and GPX4 KO clones after CRISPR/Cas9 genome editing; Figure S2. Imaging of lipid peroxidation from merged microscopic channels, red (reduced lipids) and green (oxidized lipids) from untreated controls: HCT116 WT (A) and GPX4 KO (C); and cells treated for 24 h with 10 µM of erastin: HCT116 WT (B) and GPX4 KO (D); Figure S3. Database Homepage; Figure S4. Utilities tab on FerrDb homepage.; Figure S5. Example of RNA levels correlation analysis for GPX4 and AIFM2(FSP1); Figure S6. Original Western Blot film presentation; Table S1. PCR gene expression analysis—statistical significance; Table S2. PROM2 RT-qPCR gene expression analysis—the positive control of the resistance to the erastin-induced ferroptosis cell line.
