# Supplementary material for: Compensative Resistance to Erastin-Induced Ferroptosis in GPX4 Knock-Out Mutants in HCT116 Cell Lines
Source: Pharmaceuticals (Basel). 2023 Dec 10;16(12):1710. doi: 10.3390/ph16121710 (PMC10747702; doi:10.3390/ph16121710)
Supplement: Supplementary file 1 [file pharmaceuticals-16-01710-s001.zip › pharmaceuticals-2705954-supplementary.pdf]

pharmaceuticals

Suplemmentary File

CRISPR/Cas9 mutants for GPX4 gene knock-out validation

To verify the effectiveness of the CRISPR/Cas9 deletion and changes introduced in the modified sequence of GPX4 gene, a comparison of the sequences encoded on the introduced plasmids was performed with the edited sequence located in the host genome (obtained from the manufacturer). For this purpose, primers were constructed that are located both upstream and downstream of the cleavage sites (purple sequences on Figure S1.) Carrying out the amplification reaction using designed primers allows for confirmation of changes in the tested gene sequence at a qualitative level – visualization of PCR amplicons on 1.2 % agarose (Merck); separated in 1 x TBE buffer (Merck) during horizontal electrophoresis for 30 min at 100 V, and Simply Safe stained (Eurx).

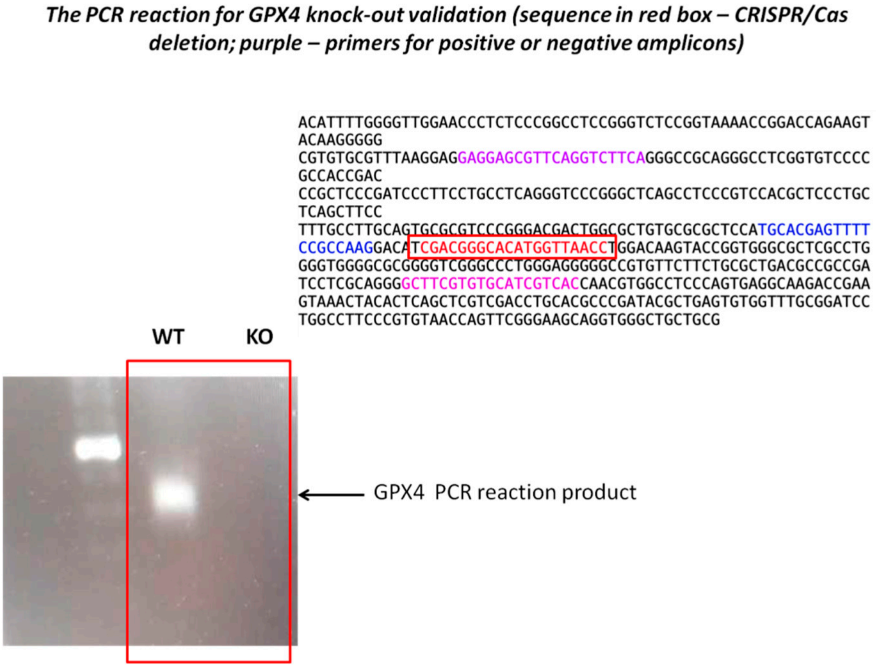

Figure S1. Procedure of GPX4 KO validation in HCT116 WT and GPX4 KO clones after CRISPR/Cas9 genome editing.

Visialisation of lipid peroxidation

To visualize lipid peroxidation in cells, an analysis was also performed using JuLI™ Stage Real-Time CHR (Cell History Recorder) from NanoEnTek. Cells were prepared on a 96-well plate as described above. After 24 hours of incubation with erastin, the plate was transferred to the JuLI™ Stage Real-Time CHR reader located in the incubator and left for analysis for 24 hours. The obtained images were processed and saved using JuLI™ Stage software from NanoEnTek.

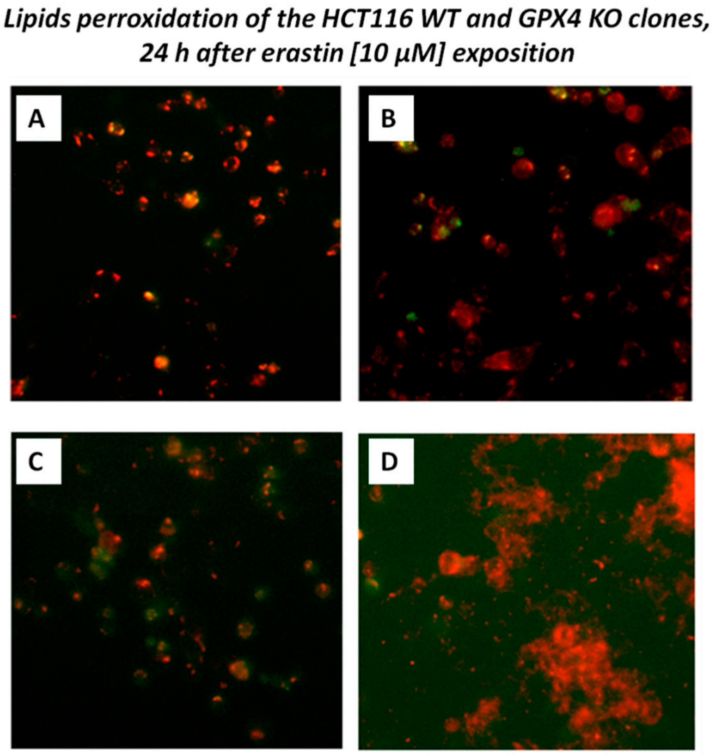

Figure S2. Imaging of lipid peroxidation from merged microscopic channels, red (reduced lipids) and green (oxidized lipids) from untreated controls: HCT116 WT (A) and GPX4 KO (C); and cells treated for 24 h with 10 µM of erastin: HCT116 WT (B) and GPX4 KO (D).

RNA correlation between genes

The analysis of RNA level correlation between genes was conducted based on data contained in the FerrDb database. FerrDb is a database encompassing information regarding regulators and markers of ferroptosis, as well as associations between ferroptosis and diseases. Annotations are generated from currently available articles on ferroptosis in the PubMed publication database. The analysis was carried out using built-in tools within the database (Utilities tab, see **Figure S4**). After selecting "correlation analysis," a new window appears where the user can choose from which database to pull data for analysis, the study acronym (in our case, it was TCGA-COAD, where COAD stands for Colon Adenocarcinoma), the number of genes to be analysed, and their respective names (**Figure S5**).

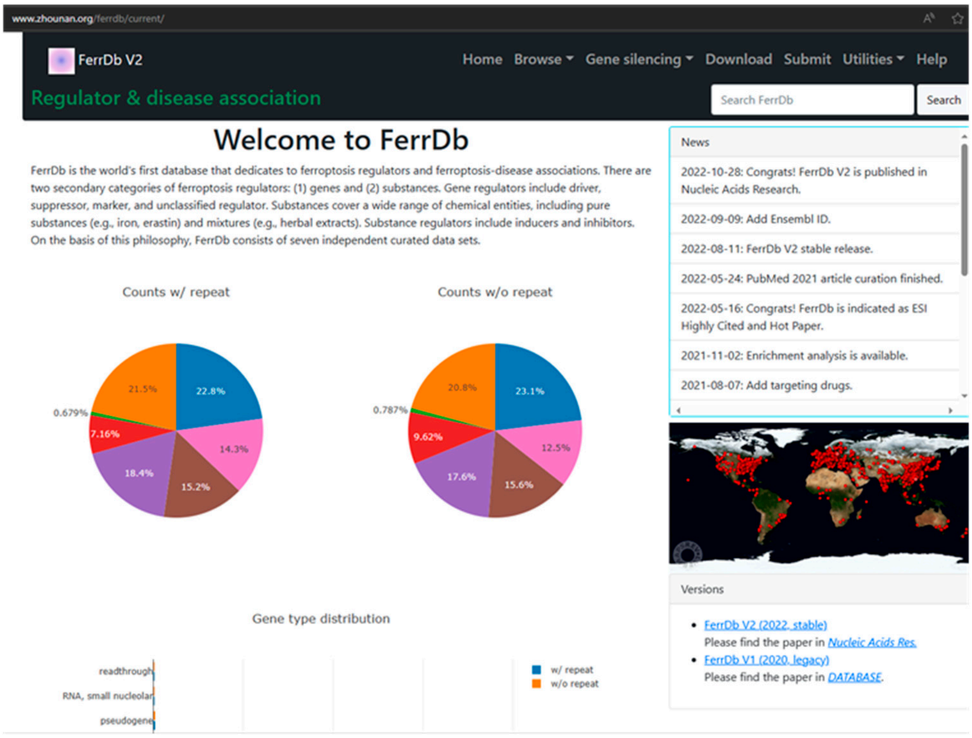

Figure S3. Database Homepage.

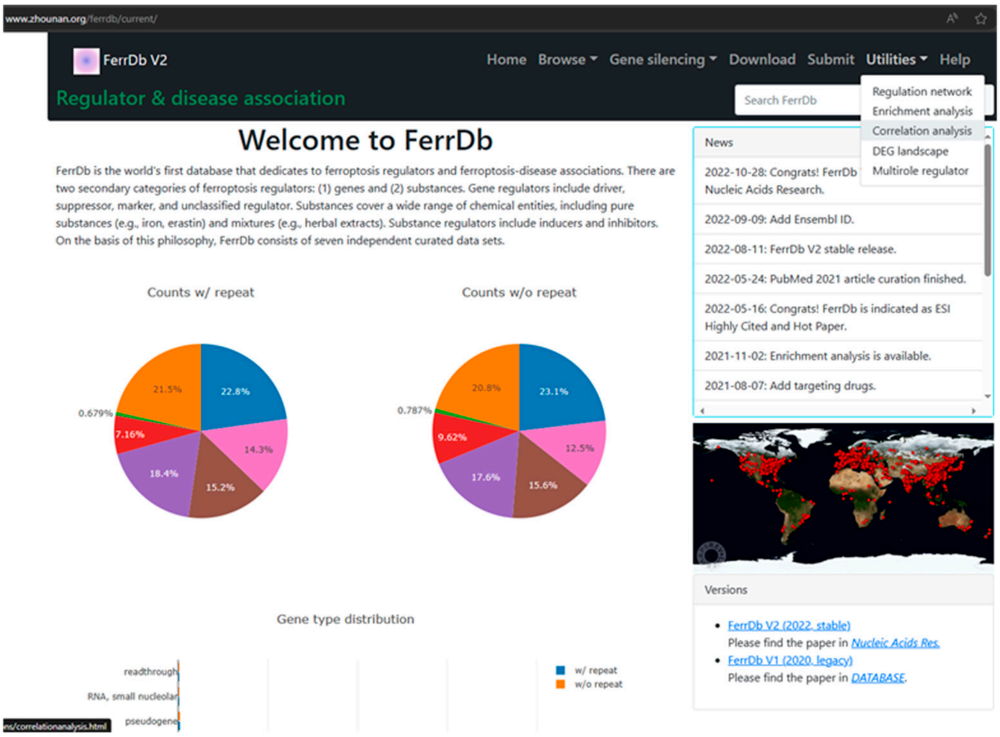

Figure S4. Utilities tab on FerrDb homepage.

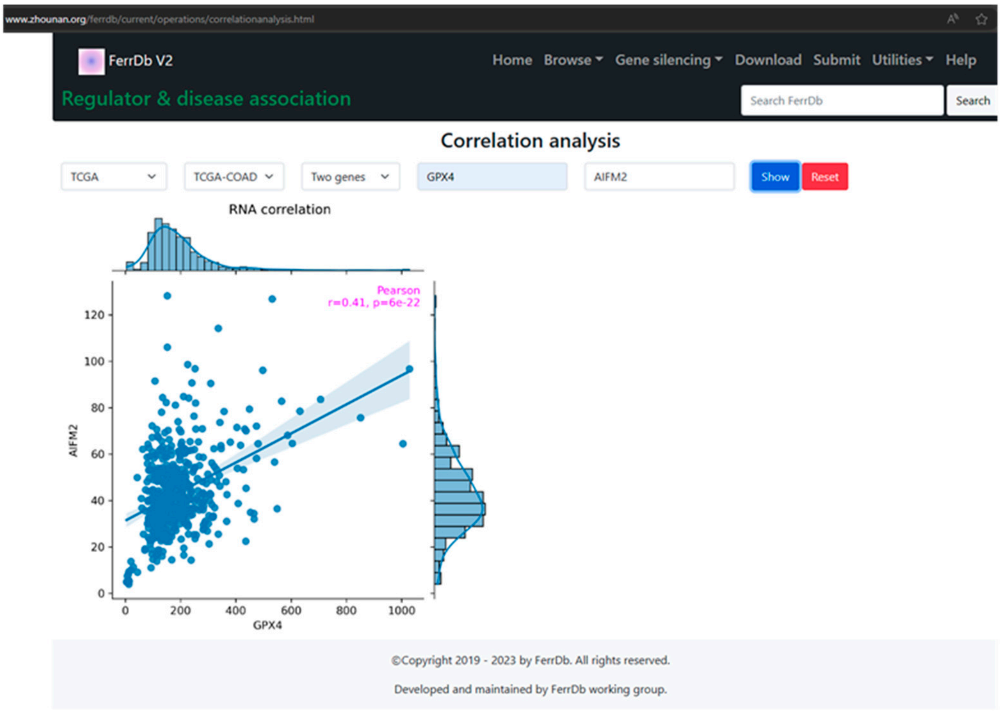

Figure S5. Example of RNA levels correlation analysis for GPX4 and AIFM2(FSP1).

Western Blot original film presentation

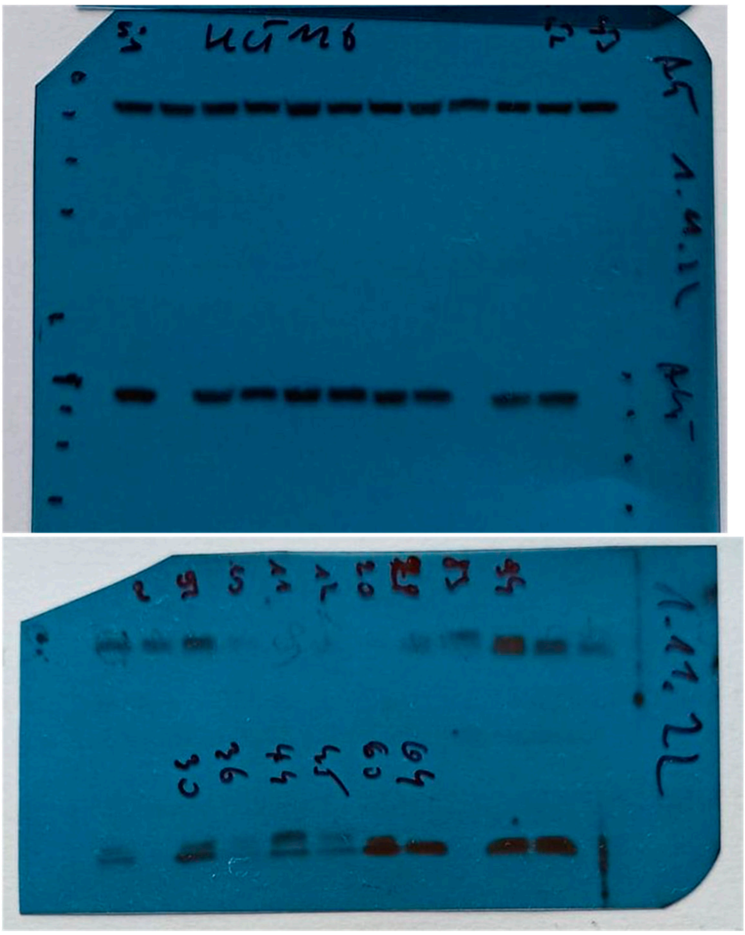

Figure S6. Original Western Blot film presentation.

Table S1. PCR gene expression analysis – statistical significance.

**HCT 116 cell line**	**WT**		**GPX4 KO**		**GPX4 POSITIVE CONTROL**	
ACSL4	CTR	ERASTIN 10 µM	CTR	ERASTIN 10 µM	CTR	ERASTIN 10 µM
average	1	0,5407526	1	1,7398498	1	0,6803623
SD	0	0,0466356	0	0,030602	0	0,0469131
test t		**0,0339632**		**0,012811**		**0,0028193**
**HCT 116 cell line**	**WT**		**GPX4 KO**		**GPX4 POSITIVE CONTROL**	
TFRC	CTR	ERASTIN 10 µM	CTR	ERASTIN 10 µM	CTR	ERASTIN 10 µM
average	1	0,3810812	1	1,1543234	1	0,4895249
SD	0	0,0913219	0	0,1320607	0	0,0921362
test t		**0,0132124**		**0,0103635**		**0,0272745**
**HCT 116 cell line**	**WT**		**GPX4 KO**		**GPX4 POSITIVE CONTROL**	
GPX4	CTR	ERASTIN 10 µM	CTR	ERASTIN 10 µM	CTR	ERASTIN 10 µM
average	1	0,9695858			1	0,4671758
SD	0	0,166051			0	0,1254838
test t		0,7169949				0,10449
**HCT 116 cell line**	**WT**		**GPX4 KO**		**GPX4 POSITIVE CONTROL**	
FSP1	CTR	ERASTIN 10 µM	CTR	ERASTIN 10 µM	CTR	ERASTIN 10 µM
average	1	0,7888401	1	1,8501584	-	n.a.
SD	0	0,1496217	0	0,2000384	-	n.a.
test t		0,2123869		**0,0342602**	-	n.a.
**HCT 116 cell line**	**WT**		**GPX4 KO**		**GPX4 POSITIVE CONTROL**	
PRDX1	CTR	ERASTIN 10 µM	CTR	ERASTIN 10 µM	CTR	ERASTIN 10 µM
average	1	0,6641726	1	1,4216603	1	0,4841059
SD	0	0,0259535	0	0,0430206	0	0,0484576
test t		**0,0019889**		**0,0034447**		**0,0029458**
**HCT 116 cell line**	**WT**		**GPX4 KO**		**GPX4 POSITIVE CONTROL**	
TRX	CTR	ERASTIN 10 µM	CTR	ERASTIN 10 µM	CTR	ERASTIN 10 µM
average	1	0,7776127	1	1,105816	1	0,5081168
SD	0	0,0718326	0	0,0166117	0	0,02237
test t		**0,0336824**		**0,0081025**		**0,0006896**
**HCT 116 cell line**	**WT**		**GPX4 KO**		**GPX4 POSITIVE CONTROL**	
PROM1	CTR	ERASTIN 10 µM	CTR	ERASTIN 10 µM	CTR	ERASTIN 10 µM
average	1	0,6999002	1	1,4000544	1	0,5877065
SD	0	0,0915564	0	0,1446081	0	0,0666175
test t		0,1327422		**0,0399374**		0,0766253

The results presented in the tables are the means from 3 biological experiments, the standard deviation and the T-test for non-treated controls; results below the threshold of $p < 0.05$ are considered statistically significant. n.a. – not analysed.

Table S2. PROM2 RT-qPCR gene expression analysis – the positive control of the resistance to the erastin-induced ferroptosis cell line.

Sample/Ct value	PROM2	RPL41
**WT control**	**36,94**	**24,55**
Erastin 5 µM	35,29	23,96
Erastin 10 µM	35,04	24,01
**GPX4 KO control**	**36,79**	**24,39**
Erastin 5 µM	36,05	24,18
Erastin 10 µM	38,49	23,75
**GPX4 positive control, control**	**36,52**	**22,84**
Erastin 5 µM	38,61	23,39
Erastin 10 µM	39,24	23,22

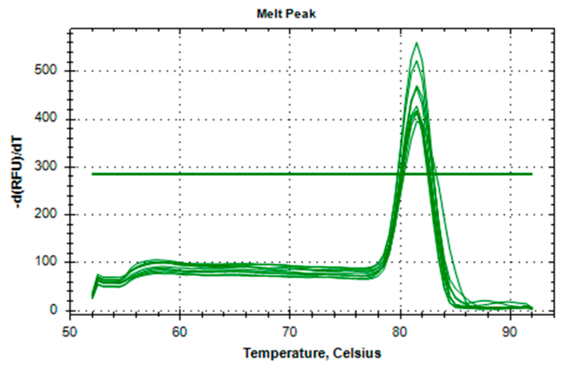

Ct – threshold cell cycle of the RT-qPCR reaction (on the left) and the melting temperature of the positive product for PROM2 expression (on the right).
